# Modulation of P1 and EGF Expression by Baicalin

**DOI:** 10.3390/ijms14010146

**Published:** 2012-12-20

**Authors:** Yanli Meng, Jinhai Huo, Weihong Lu, Xin Wang, Junwei Zhang, Weiming Wang

**Affiliations:** 1 Heilongjiang Academy of Traditional Chinese Medicine, Harbin 150036, China; E-Mails: songxy60@yahoo.cn (Y.M.); jinhaihuo@126.com (J.H.); keaidyuxi@163.com (X.W.); laozhang-101@126.com (J.Z.); 2 School of Food Science and Engineering, Harbin Institute of Technology, Harbin 150090, China; E-Mail: lwh@hit.edu.cn

**Keywords:** *M. pneumoniae*, Baicalin, EGF, P1

## Abstract

*Mycoplasma pneumoniae* (*M. pneumoniae*) is increasingly recognized as a major cause of acute respiratory tract infections. Today, macrolides are used in the primary treatment of *M. pneumoniae* infection. However, with the increasing prevalence of strains resistant to macrolides, as well as reports of toxicity and adverse side effects, it is necessary to develop an alternative therapeutic agent. A compound recipe—Qinbaiqingfei pellets (Qinbai)—have already been approved in China as the first effective traditional Chinese medicine to be used against *M. pneumoniae*. Herein, we characterize the mechanism by which Qinbai interacts with *M. pneumoniae* and lung epithelial cells. The fact that Baicalin is the key component of Qingbai leads us to believe its study is important to elucidating the mechanism of the action of Qinbai. In this study, we describe the complex impact of Baicalin on the adhesin protein P1 of *M. pneumoniae* and on the expression of epidermal growth factor (EGF) in BALB/c mice and A549 cells infected with *M. pneumonia*. We draw the conclusion that Baicalin not only cured *M. pneumoniae* infection by inhibiting P1 expression, but also enhanced the repair of lung epithelial cells by upregulating EGF. Finally, we demonstrate that Baicalin plays a role in Qinbai treatment.

## 1. Introduction

*Mycoplasma* is a large genus, which includes *Mycoplasma pneumoniae*, and members characteristically have a small size and an AT-rich genome. *M. pneumoniae*, which can cause infection of the respiratory tract and mycoplasma pneumonia, has emerged as the most frequent pathogen affecting children of all ages, and is responsible for 20% of all cases of community-acquired pneumonia every year [1,2]. Since *M. pneumoniae* lacks a cell wall, it is unharmed by many antibiotics such as penicillin. Macrolides have historically been used to treat *M. pneumoniae* infections in adults and children because it possesses anti-inflammatory properties as well as potent antibacterial activity. However, during the past several years, clinical and experimental research has shown that repeated treatment with azithromycin can lead to adverse consequences, such as toxicity, side effects and drug resistance, which have been associated with clinical treatment failure [3,4]. Recently, a report from Japan found 10% to 33% of *M. pneumoniae* isolates are no longer susceptible to macrolides and may not respond to treatment with these drugs [5,6]. Furthermore, a study from Shanghai, China, has reported that 39 of 50 *M. pneumoniae* isolates tested were macrolide resistant [7], illustrating its limited use. In addition, repeated infection can often lead to respiratory structure damage. Thus, it is necessary to develop a novel clinical therapeutic that not only combats *M. pneumoniae* infection, but can also enhance the repair of lung epithelial cells.

Qinbai, which is extracted from plants such as *Scutellaria Baicalensis Georgi*, is the first effective traditional Chinese medicine to be approved by the Chinese government for the clinical treatment of infections with sensitive and resistant strains of *M. pneumoniae*. Clinical evidence suggests that Qinbai not only has an anti-*M. pneumoniae* activity, but also has a strong protective effect on lung epithelial cells. Baicalin, isolated from the plant *Scutellaria Baicalensis Georgi*, is the main component of Qinbai. *Scutellaria Baicalensis Georgi* has been an important herb in China for thousands of years, and shows great pharmacological potential in the treatment of inflammation, cancers and viral diseases such as H1N1 and HIV-1 [8,9]. However, until now, the mechanism by which this drug opposes *M. pneumoniae* and protects lung epithelial cells has not been investigated. Since Baicalin is a key part of Qinbai, we hypothesize that in addition to its anti-*M. pneumoniae* properties, Baicalin also plays a key role in the protection of lung epithelial cells.

Recently, growing evidence has shown that a key element of *M. pneumoniae* infection is its adherence to respiratory epithelial cells by a terminal structure; this is a membrane-bound protein structure consisting of the major surface P1 adhesin molecule, which plays a key role in adhesion, gliding motility movement and cell division [10,11]. After the epithelial cells that serve as physical barriers are damaged, proliferative epithelial cells migrate to cover denuded areas and restore their functions. Activation of the EGF plays a key role in regulating cell survival and apoptosis, as well as initiating motility in poorly healing wounds [12].

In this study, we employ *M. pneumonia*, BALB/c mice and a human-derived epithelial cell line, A549 type II pneumocytes, to study the effects of Baicalin treatment. We focused on the expression levels of P1 and EGF, as we hypothesized that Baicalin is likely to inhibit the adhesion of *M. pneumoniae* by decreasing expression of P1 and promote lung epithelial cell proliferation by increasing EGF expression. To test these hypotheses, we have compared P1 expression at the levels of transcription and translation. Additionally, we attempted to determine how Baicalin regulates EGF, with particular attention paid to EGF mRNA and protein expression in BALB/c mice and A549 cells. Our observations on its inhibition of the adhesion protein P1, and it ability to upregulate EGF, are of significant therapeutic importance.

## 2. Results and Discussion

*Scutellaria Baicalensis Georgi* and its constituent molecule, Baicalin, are traditional Chinese medicines that have been investigated for their ability to prevent viral and bacterial diseases for thousands of years. However, the anti-*M. pneumoniae* property and the pro-epithelial repair effects of Baicalin and *Scutellaria Baicalensis Georgi* could not be explained until now. In this study, we demonstrate that Baicalin plays a pivotal role in the therapeutic effect of Qinbai, not only in the killing of *M. pneumoniae*, but also in the restoration of lung epithelial cells. Our research strategy involved investigating the protein and mRNA expression of the P1 surface adhesin and EGF. This is, to the best of our knowledge, the first study showing that Baicalin possesses the ability to regulate P1 and EGF expression, and also provides an additional potential mechanism to explain the activation of Baicalin.

### 2.1. Pathological Changes in the Lung Tissue

**Figure 1:**
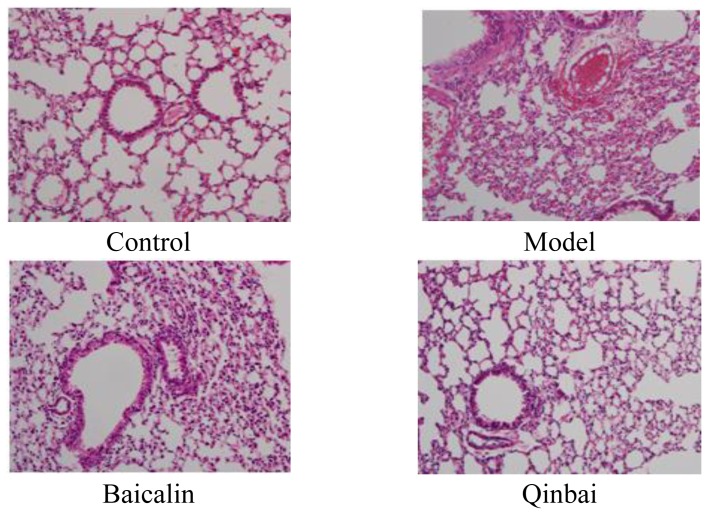

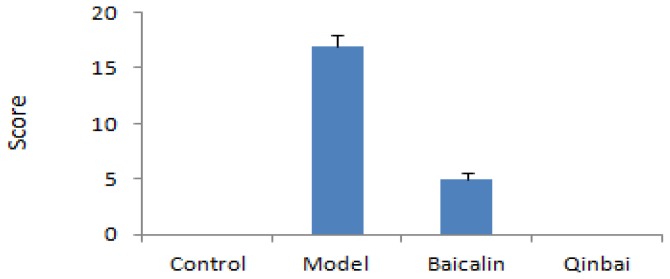
The pathological examination of the lungs of BALB/c mice and the histology scores revealed marked differences between groups. After mice were infected with *Mycoplasma pneumoniae* for 3 days, they were treated with 16 μg/mL Baicalin and 100 μg/mL Qinbai for 6 days. Experiments were repeated twice.

Hematoxylin and eosin (HE) staining was first performed to observe the pathological changes occurring in the lung tissue (Figure 1). Each histopathological change was scored on a scale from 0 (no change) to 26 (maximum inflammation) being based on peribronchiolitis, perivasculitis, interstitial pneumonitis, and alveolitis (Figure 1). The score demonstrated significant increases for the model group compared with controls. In the model group, we found bronchial epithelial desquamation, peribronchovascular interstitial thickening, and alveolar collapse; in addition, the capillaries in the alveolar walls congested with many red blood cells. Baicalin-treated mice had partial bronchial epithelial desquamation. The Qinbai-treated group was similar to the control group (HE staining × 200).

### 2.2. Study of the Anti-*M. pneumoniae* Effects of Baicalin

**Figure 2:**
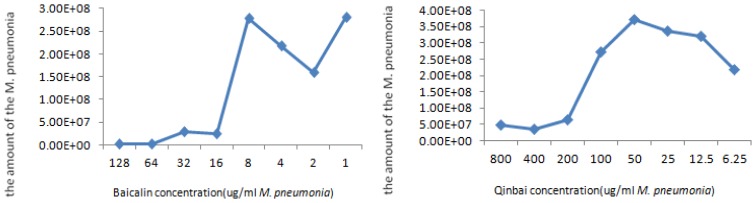
*M. pneumoniae* was incubated at 37 °C with various concentrations of Baicalin and Qinbai by the serial double dilution method. MIC was 16 μg/mL for Baicalin and 100 μg/mL for Qinbai at which the metabolism of the organisms was inhibited and lack of color change was observed. Resultant mixtures are measured by real-time quantitative PCR. Baicalin and Qinbai demonstrated a reasonably good activity against *M. pneumoniae*. Our study suggests that Baicalin may have great potential in clinical studies.

*M. pneumoniae* is responsible for a variety of damage in humans by a distinct polar structure that includes the major adhesin protein P1, which forms an intimate relationship to epithelial cells that mediates attachment to host cells. This molecule also mediates gliding motility by promoting movement of *M. pneumoniae* on airway surfaces to allow easier contact with host receptors and to seek refuge from the mucociliary escalator [13–15]. The data presented herein provide essential information on how Baicalin inhibits *M. pneumoniae* during the infectious process. A positive correlation between Baicalin and P1 levels was observed in this study, as decreased P1 expression is detected after *M. pneumoniae* is treated with Baicalin. Since P1 is thought to be necessary for the adherence of *M. pneumoniae* to ciliated epithelium within the respiratory tract, we draw the conclusion that Baicalin prevents *M. pneumoniae* infection through the inhibition of adhesin protein expression. The result is that the minimal inhibitory concentration (MIC) of Baicalin is 16 μg/mL (with a range of 1–128 μg/mL) and presents with greater levels of bactericidal activity than Qinbai, which has a MIC of 100 μg/mL (and a range of 6.25–800 μg/mL), suggesting that *M. pneumoniae* is more sensitive to Baicalin (Figure 2). Interestingly, though this was not the focus of the current study, the MIC of Baicalin demonstrates a standard of given medicines.

**Figure 3:**
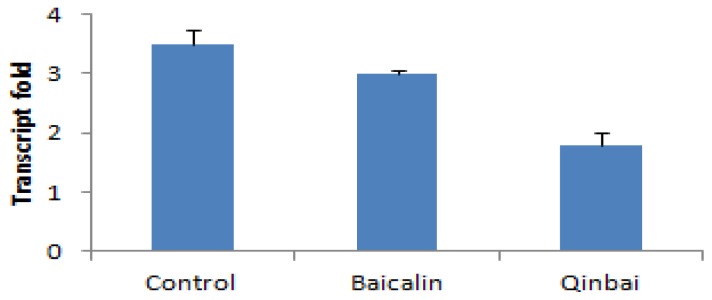
P1 mRNA expression was inhibited by treatment with 16 μg/mL Baicalin and 100 μg/mL Qinbai. Real-time quantitative PCR was performed as detailed in the Materials and Methods. Experiments were repeated three times. Data are presented as mean ± SD, *p* < 0.01.

**Figure 4:**
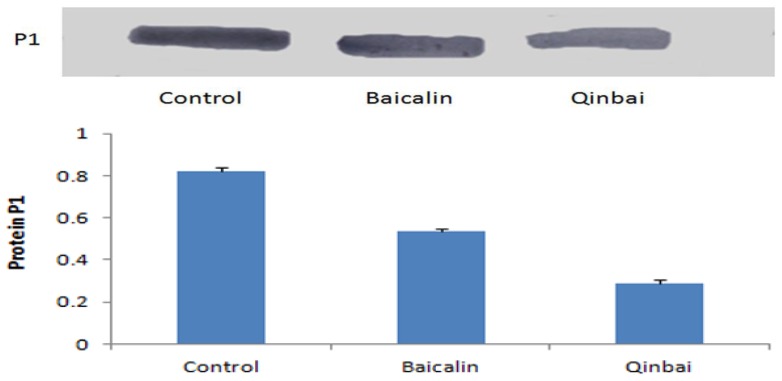
Expression of the P1 protein was assessed by immunoblot. Baicalin and Qinbai depressed P1 protein expression. Experiments were repeated twice. Data are presented as mean ± SD, *p* < 0.01.

We wanted to determine if P1 expression at the transcription and translation level is altered after treatment with the MICs of Baicalin (16 μg/mL) and Qinbai (100 μg/mL) for 3 days; therefore, real-time quantitative PCR (Figure 3) and Western blotting (Figure 4) were carried out. The mRNA and protein expression levels were compared in the different groups, and the results suggested that Baicalin and Qinbai significantly downregulated P1 gene transcription and protein translation, especially in the latter group.

### 2.3. Effects of Baicalin on EGF mRNA and Protein Expression in BALB/c Mice

Next we attempted to determine if the recovery of lung epithelial cells damaged by *M. pneumoniae* infection is associated with EGF expression. It would be important to know prior to its use in therapy if Baicalin can enhance EGF to encourage epithelial cell proliferation. EGF is the most potent of the epithelial growth factors. Elevated levels of EGF may improve epithelial proliferation, which dramatically affects the cellular functions [16]. In wound healing, EGF is a critical factor released by platelets at the site of injury, and it stimulates epidermal cell proliferation and migration [17]. EGF is also capable of reducing scarring by preventing excessive wound contraction [18]. We used PCR, immunoblotting and immunofluorescence to investigate EGF mRNA and protein expression; changes in transcriptional and translational levels were measured, which is important because mRNA and protein analysis make it possible to study drug mechanisms. We found that groups treated with Baicalin had significantly increased EGF expression relative to the model group. The high EGF expression found in the lung epithelial cells may play a key role in the recovery of these cells. These results may provide an explanation of why Baicalin has a distinct effect on lung epithelial cell proliferation.

**Figure 5:**
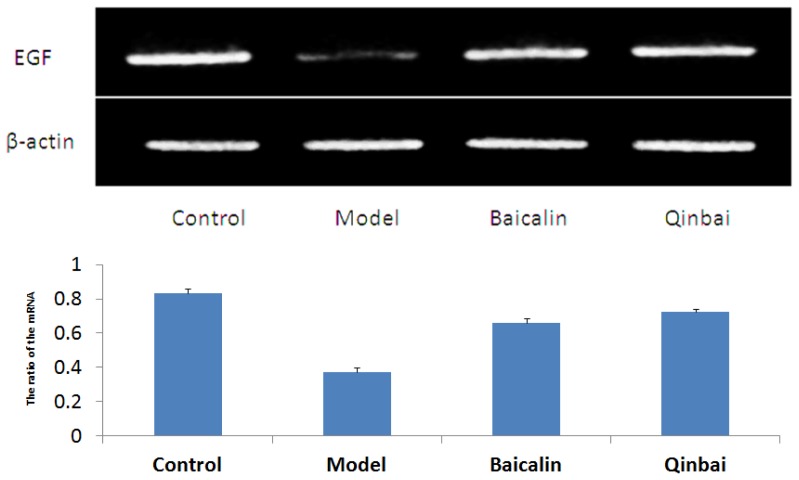
EGF mRNA expression was increased in BALB/c mice treated with Baicalin and Qinbai. After mice were infected with *M. pneumoniae* for 3 days, they were treated with 16 μg/mL Baicalin and 100 μg/mL Qinbai for 6 days. Experiments were repeated three times. Data are presented as mean ± SD, *p* < 0.05.

**Figure 6:**
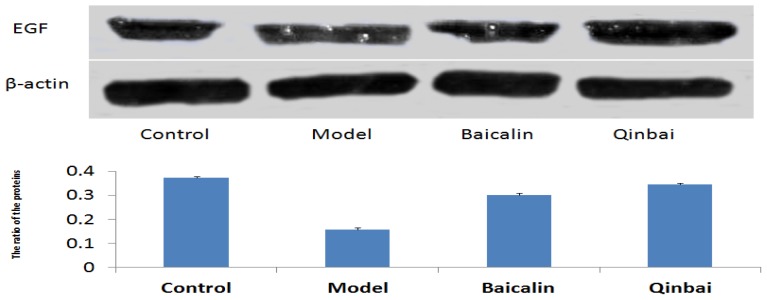
Baicalin and Qinbai had a significant effect on EGF protein expression. Protein extracted from BALB/c mice treated with Baicalin or Qinbai was analyzed by Western blot with an antibody against EGF. Experiments were repeated twice. Data are presented as mean ± SD, *p* < 0.05.

**Figure 7:**
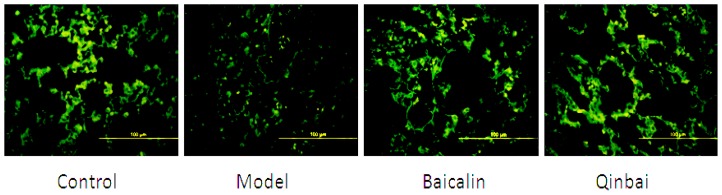
The treatment of BALB/c mice with Baicalin or Qinbai led to an increase in EGF protein expression according to immunofluorescence results. Experiments were repeated twice.

To address whether EGF mRNA expression is induced by Baicalin and Qinbai, we analyzed mRNA and protein expression of the mice lungs by reverse transcription-PCR (Figure 5), immunoblotting (Figure 6) and immunofluorescence (Figure 7). RT-PCR analysis indicated that both agents significantly stimulated EGF mRNA expression. The treatment with Baicalin or Qinbai also led to an increase in EGF protein expression, especially in the Qinbai group.

### 2.4. Effects of Baicalin on EGF mRNA and Protein Expression in A549 Cells

**Figure 8:**
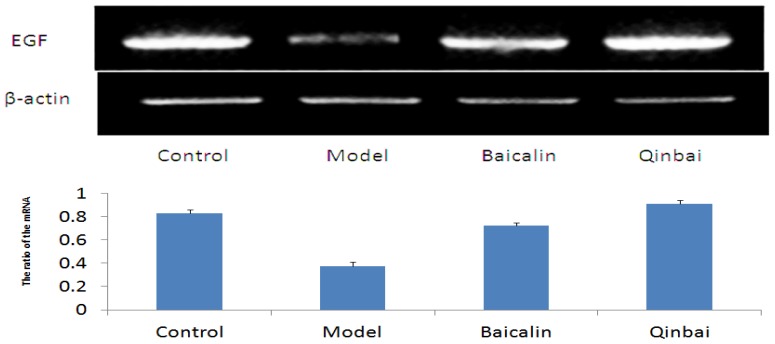
We performed reverse transcription-PCR to analyze the levels of EGF mRNA. After A549 cells were infected with *M. pneumoniae* for 4 h, cells were treated with 16 μg/mL of Baicalin and 100 μg/mL of Qinbai for 3 days at 37 °C. EGF expression in A549 cells was increased by Baicalin and Qinbai. Data are presented as mean ± SD, *p* < 0.05.

**Figure 9:**
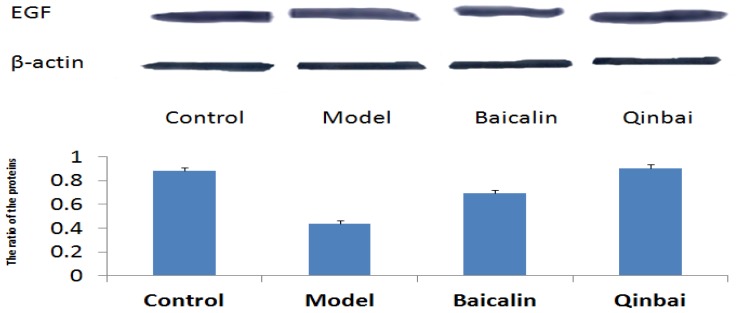
The treatment of cells with Baicalin or Qinbai led to an increase in EGF protein expression according to immunoblotting. Data are presented as mean ± SD, *p* < 0.05.

**Figure 10:**
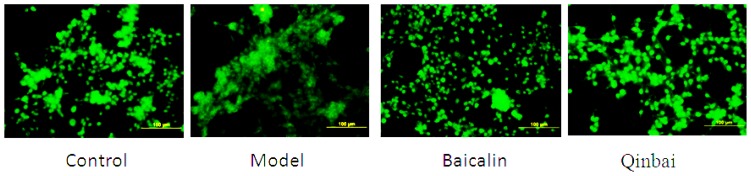
Immunofluorescence was used to analyze EGF protein expression. Experiments were repeated twice.

To further elucidate the mechanism by which Baicalin affects EGF expression, we harvested A549 cells that were infected with *M. pneumoniae* and analyzed EGF mRNA and protein expression. Our results indicated that EGF mRNA expression was upregulated in the treated groups (Figure 8). We also analyzed the protein profiles of four samples with the use of immunoblotting (Figure 9). Compared with the control group, Qinbai greatly increased the protein levels of EGF, and Baicalin-treated cells also showed an increase. We also analyzed EGF protein levels with immunofluorescence (Figure 10), and found that EGF expression was increased in the treated group.

Although our study suggests that the induction of EGF expression by Baicalin is one mechanism by which this molecule is beneficial, further studies are needed to explore the therapeutic potential of Baicalin. EGF is preferentially co-expressed with EGFR in malignant cases. Activation of EGFR and EGF has been found to induce proliferation through the upregulation of various signaling pathways, including PI3K/Akt, STAT and Ras/Raf/MAPK [19], which is one of the most important pathways regulating EGF expression. Therefore, additional studies are needed to understand whether Baicalin induces EGF by stimulating factors in these signaling pathways.

## 3. Materials and Methods

### 3.1. Reagents

Trizol was purchased from Invitrogen (Carlsbad, CA, USA). A real-time quantitative PCR kit used for *M. pneumoniae* studies was purchased from Da An Gene (Guangzhou, China). Reverse transcription reagents and the real-time quantitative PCR kit were purchased from Takara (Dalian, China). A one-step RT-PCR kit was purchased from Tiangen (Beijing, China). Mouse anti-EGF and mouse anti-β-actin antibodies were purchased from Abcam (Cambridge, MA, USA). The goat anti-P1 antibody was purchased from Santa Cruz (Delaware Avenue, CA, USA). Baicalin was purchased from the Heilongjiang Institute for Drug Control.

### 3.2. *M. pneumoniae* and Cell Culture

After the *M. pneumoniae* (ATCC 15531) purchased from American Type Culture Collection was cultured in serum for 24 h, *M. pneumoniae* strain was grown at 37 °C in PPLO medium supplemented with yeast extract, glucose, penicillin, and 20% fetal calf serum. Growing *M. pneumoniae* were divided into control and experimental groups; the latter group was treated with 16 μg/mL Baicalin or 100 μg/mL Qinbai (MIC) for 3 days. Following these procedures, RNA and protein were extracted from the *M. pneumoniae* cells. A549 cells, obtained from the Laboratory Center of the Harbin Tumor Hospital, were cultured in 1640 medium with 10% fetal calf serum. 10^5^ cells were infected with 10^6^ CCU of *M. pneumoniae* for 4 h, and the medium was removed along with unbound *M. pneumoniae*. The infected cells were divided into a control group, a model group, and groups that were treated with 16 μg/mL of Baicalin and 100 μg/mL of Qinbai at 37 °C for 3 days. Cells were collected to extract mRNA and protein. After 10^5^ cells were treated on slides placed in 6-well plates for 3 days, immunofluorescence was performed.

### 3.3. Animals

BALB/c mice were obtained from the Animal Laboratory Center of Harbin Tumor Hospital and maintained in a GLP room at 25 °C and 60% humidity. The mice were randomly divided into control, model, and treated groups (*n* = 10). After model and treated groups were infected with 20 μL of 10^6^ CCU *M. pneumoniae* for 3 days, mice in the treated group were gavaged once daily with 80 mg/kg/d Baicalin and 2.3 g/kg/d Qinbai for 6 days, while mice of the control group and model group were given an equal volume of saline. Lung samples were collected to detect the mRNA and protein levels of EGF, and a section of lung was processed for immunofluorescence microscopy and HE stain.

### 3.4. Minimal Inhibitory Concentration

The minimal inhibitory concentration (MIC) was considered the dose of drug at which the metabolism of the organisms was inhibited and lack of color change was observed. The MIC of Baicalin and Qinbai were determined by the double dilution method. One hundred twenty-eight micrograms of Baicalin and 400 μg of Qinbai were added to 1 mL of medium that included 100 μL 10^6^ CCU *M. pneumoniae* and serial double dilutions of the drugs (range of Baicalin: 128 to 1 μg/mL; range of Qinbai: 800 to 6.25 μg/mL) were prepared. The resultant mixtures were incubated at 37 °C and not collected until the phenol red turned to the yellow of the control, and were then analyzed by real-time quantitative PCR (Bio-rad, Richmond, CA, USA).

### 3.5. HE Stain

The lungs of animals were removed, placed in 10% formalin for less than 24 h, and then embedded in paraffin. Serial tissue sections (5 μM thick) were deparaffinized, hydrated in xylene and graded alcohol solutions, and stained with HE using a histopathologic inflammatory scoring system as described previously [20].

### 3.6. Real-Time Quantitative PCR and Reverse Transcription-PCR Analysis

*M. pneumoniae* genomic DNA was isolated by 10 min incubation in 100 °C boiling water. The real-time quantitative PCR program was as follows: an initial step of denaturation 95 °C for 30 s, followed by 40 cycles of 95 °C for 5 s and 60 °C for 30 s. Total RNA was extracted from cell lines, the lungs of mice and *M. pneumoniae* using the Trizol RNA isolation reagent according to the manufacturer’s instructions. The total RNA was quantified and EGF mRNA was amplified with a one-step reverse transcription-PCR kit purchased from Bio-Rad ((Bio-rad, Richmond, CA, USA). The following thermocycling program was used: 50 °C for 30 min, an initial 94 °C denaturation step for 2 min, followed by 40 cycles of denaturation at 94 °C for 1 min, primer annealing at 55 °C for 1 min and extension at 65 °C for 2 min. The amplified cDNA fragments were electrophoresed through a 1% agarose gel and visualized under an imaging densitometer (Bio-rad, Richmond, CA, USA). Analysis of *M. pneumoniae* P1 expression was performed by real-time quantitative PCR reaction (Bio-rad, Richmond, CA, USA). The thermal profile was as follows: 1 cycle of 25 °C for 5 min, 45 °C for 60 min and 70 °C for 5 min, followed by an initial step of denaturation 95 °C for 30 s and 40 cycles of 95 °C for 5 s and 60 °C for 30 s. H-EGF-forward primers 5′-TAAGGCGTGGTCAGCATC-3′ and reverse primers 5′-GAAACCCGACGAACAGAG-3′. M-EGF-forward primers 5′-GTCACCCACAGAAACAAT-3′ and reverse primers 5′-AGGAGCGAACCTACTAAA -3′. H-β-actin-Forward primers 5′-AGAGATGGCC ACGGCTGCTT-3′ and reverse primers 5′-GCCACAGGACTCCATGCCCA-3′. M-β-actin-forward primers 5′-GACGGCCAGGTCATCACTATTG-3′ and reverse primers 5′-AGGAAGGCTGGAAAA GAGCC-3′. P1-forword primers 5′-CCTTGTGAATTTGCTCAG-3′, reverse primers 5′-GTCAGCTGC AGTTAGAAA-3′ and probe (5′-AATGATCTCGCCAACGCTCCT-3′).

### 3.7. Immunofluorescence Analysis

Slides of cells and mice lungs were fixed with 4% paraformaldehyde and were then blocked with 8% BSA for 2 h. Slides were incubated overnight at 4 °C with primary mouse monoclonal anti-EGF antibodies. The slides were then immunostained with their corresponding FITC-conjugated secondary antibodies in the dark for 2 h at room temperature. Slides were visualized and photographed under a fluorescent microscope (Olympus, Tokyo, Japan).

### 3.8. Western Blot Analysis

The total protein of cells and mice lungs was extracted with lysis buffer containing a protease inhibitor (Roche, Mannheim, Germany), and the protein concentration was measured with a protein assay reagent kit (GE, Piscataway, NJ, USA). Equal amounts of protein from each sample were electrophoresed through a 12% SDS–PAGE gel and transferred onto a PVDF membrane. Membranes containing protein were incubated overnight at 4 °C with mouse monoclonal anti-EGF. Membranes were then incubated with secondary antibody for 90 min at room temperature. Bands were visualized with chemiluminescence reagents.

### 3.9. Statistical Analysis

Analyses of the reverse transcription-PCR and Western blot results were performed using Gel-Pro 4.0 software (Media Cybernetics, Rockville, MD, USA). Data are presented as mean ± SD. Differences were considered significant at *p* < 0.05.

## 4. Conclusions

Our results demonstrate that Baicalin not only inhibits the adhesion of *M. pneumoniae* by decreasing P1 expression, but also helps to restore epithelial cells by increasing EGF expression. We showed that the effect of Baicalin on *M. pneumoniae* was quite similar to that of Qinbai, and led to similar functional outcomes. However, Qinbai showed a stronger effect, which suggests that Qinbai may have a more complex mechanism of action. Elucidating the differences between Qinbai and Baicalin is useful in understanding complementary superiority of traditional Chinese medicine. These findings encourage future work exploring the other component of Qinbai, and may motivate the study of the changes in signaling pathways induced by these drugs.
